# Perception and misperception of social corrections: the case of challenging violations of gender equality and social inclusion on social media

**DOI:** 10.1098/rsos.252547

**Published:** 2026-11-11

**Authors:** Rabab Ali Abumalloh, Osama Halabi, Ala Yankouskaya, Raian Ali

**Affiliations:** ^1^ Department of Computer Science and Engineering, Qatar University, Doha, Qatar; ^2^ Department of Computing, Atlantic Technological University, Letterkenny, Ireland; ^3^ School of Psychology, Bournemouth University, Poole, UK; ^4^ College of Science and Engineering, Hamad Bin Khalifa University, Doha, Qatar

**Keywords:** gender equality, social inclusion, social correction, misperception

## Abstract

Social media platforms enable both gender equality and social inclusion (GESI) advocacy and GESI violations, such as hate speech and harassment. Challenging such violations through social correction by active users is essential, yet little is known about whether users accurately perceive others' attitudes towards this behaviour across cultural contexts. This cross-sectional study, based on an online survey of 325 UK and 312 Arab participants, examines self-reported attitudes and perceptions of others' attitudes towards three barriers to social correction: Relationship Consequences, Negative Impact on others and Futility, using linear mixed modelling. Findings indicate a discrepancy between self-reported attitudes and perceptions of others' attitudes, indicating a misperception. In the UK sample, misperceptions relate to Relationship Consequences and Futility, with participants expecting greater relational costs and less behavioural change when challenging others than when being challenged themselves. In the Arab GCC sample, participants perceived the challenged person as more negatively affected than they believed they themselves would be, though this pattern was less consistent across individual measures. These findings suggest that integrating social norm nudges into social media design could correct misperceived norms and empower users to challenge GESI violations. Limitations include sample representativeness and self-reported data; future research should use broader samples and qualitative methods.

## 1. Introduction

Gender equality and social inclusion (GESI) concepts aim to establish equitable rights and access to resources and opportunities for individuals across all genders, ethnicities and socio-economic statuses [1]. The term ‘gender equality’ typically does not imply treating men and women identically, but rather ensuring they have equal access to resources and opportunities [2]. Social inclusion and its opposite, social exclusion, can be viewed as either a personal characteristic or a social status [3]. Social inclusion is viewed as both a policy process involving strategies to integrate marginalized groups and a policy objective aimed at achieving equal opportunities and full societal integration for all individuals [3]. Promoting these concepts on social media necessitates a thorough comprehension of the psychographic and cultural traits of online users and possibly designing social media differently to accommodate diversity.

The integration of social media platforms into daily life has introduced new modes of interaction, enabling the rapid sharing of information and the facilitation of community building and online networking. The emerging modes of interaction through social media can contribute to reducing the gender gap across various fields and enhancing social inclusion [4]. Conversely, the widespread and accessible use of social media has facilitated various forms of online violations such as misinformation [5] and cyber-bullying, including insults, harassment and cyber threats [6]. These violations often manifest as sending offensive messages or sharing inappropriate posts via social media and online platforms [7]. Such violations intensify as individuals, feeling less constrained online, exhibit increased aggression owing to a heightened sense of anonymity and invisibility [8]. This issue has a range of detrimental effects on the victim's physical and mental well-being, including anxiety, diminished self-esteem, sensations of isolation, sleep disturbances and depressive symptoms [9]. Understanding who intervenes in such violations and what shapes their decision to do so is therefore as important as understanding the violations themselves.

Online violations are intensified by the considerable influence of a small, toxic group that predominantly shapes the nature of online discussions. This phenomenon encourages users to generate content to expand their audience, thereby distorting public perceptions of social norms [10]. Based on findings from a longitudinal study of 929K accounts posting toxic comments on Reddit over 18 months, Kumar *et al*. [11] observed that while only 3% of active accounts engage in toxic behaviour, these accounts generate 33% of all social media content. The design of social media platforms plays a crucial role in this context. For instance, in the study by Milli *et al*., the authors find that the engagement-based ranking system of Twitter (now X) promotes emotionally intense and hostile content aimed at out-groups. The visible and amplified presence of hostile content may lead users to believe that intolerance is more widespread than it actually is, raising the question of how individuals and platforms alike might respond to correct this distorted picture.

In literature, two primary avenues have been investigated concerning addressing such behaviours: coping strategies employed by victims and corrective measures undertaken by diverse stakeholders. Regarding victims, numerous studies have delved into their coping mechanisms in response to such violations, including confrontation, avoidance/ignoring, seeking support and retaliation [12]. On the other hand, corrective measures can be categorized as providing technological solutions, such as employing AI-based content moderation [13], and human interventions from multiple stakeholders, including parents of victims, decision makers in educational institutions and communities [14], and other users who observe these violations [15]. However, existing research has largely focused on victims' coping strategies [16], technological solutions [17] and the occurrence of stakeholder intervention itself, including bystander correction [18]. Comparatively little attention has been paid to how individuals' own attitudes and their perceptions of others' attitudes towards challenging online violations diverge or whether such misperceptions shape stakeholders' willingness to intervene in the first place. This gap is particularly pronounced in cross-cultural contexts, where misperceptions may manifest differently across societies.

Challenging individuals who violate GESI concepts on social media, by other active users, can be an effective approach to address such harmful behaviour and foster a more respectful and inclusive online environment. Scholars have examined individuals' readiness to voice their opinions on social media, drawing from Noelle-Neumann's Spiral of Silence theory [19]. This theory suggests that people's perceptions of the prevailing beliefs and opinions in society influence their tendency to speak out or stay silent in social settings [19]. Three key barriers may discourage users from challenging GESI violations. First, barrier is Relationship Consequences, which refer to users' perceptions that challenging others may damage their relationships, such as being seen as hostile or causing reduced future interactions [20]. Second, Negative Impact on others refers to the concern that the person being challenged may feel offended or embarrassed [21]. Third, Futility reflects a sense that challenging violations will yield no meaningful change [22]. The perception of futility, which suggests that one's actions will have little impact on changing someone's opinion, can discourage individuals from taking corrective measures [23]. Whether these barriers reflect accurate assessments of others' reactions or rather misperceived social norms is a central question that connects individual behaviour to broader theories of social norms and collective perception. Building on this, we aim to answer the following research questions:

— RQ1: Is there a disparity between individuals' self-reported attitudes and their perceptions of others' attitudes towards being challenged when violating GESI principles on social media with regard to the following:
  (i) RQ1.1: Relationship consequences, that is, impacting the relationship between the challenging and challenged users;
  (ii) RQ1.2: Negative Impact on the one being challenged; and
  (iii) RQ1.3: Futility, that is, the pointlessness or uselessness of challenging.
— RQ2: Are there cultural differences between the UK and Arab participants in terms of their self-reported attitudes and their perceptions of others' attitudes towards being challenged when violating GESI principles?

Accordingly, this study has two objectives: first, to examine whether users accurately perceive others' attitudes towards challenging GESI violations on social media; and second, to identify how any misperceptions differ between UK and Arab GCC cultural contexts.

## 2. Theoretical grounds

Social norms are the implicit guidelines that a social group either consciously or unconsciously enforces, reflecting how its members are expected to behave [24]. Social norms shape expectations and encourage conformity by providing a framework through which individuals interpret and evaluate the behaviours and attitudes of others. The perceptions of social norms within groups are referred to as collective norms, which influence individuals' views and, consequently, their behaviours [25]. People's understanding of social norms may not necessarily align with the genuine norms (such as the real intentions or actions of others). Misperception describes the gap between a person's actual attitude or behaviour and the assumptions about others' attitudes or behaviours [26]. As a result of a commonly misperceived collective norm, individuals may mistakenly believe that the behaviours and attitudes of others differ from their own, even though they are actually quite similar [27]. According to Gurgun *et al*. [28], individuals on social media often overestimate the negative reactions they may face when challenging misinformation, revealing a disconnect between their perceptions and the actual responses in such interactions.

A closely related and well-documented form of misperception is pluralistic ignorance, which occurs when people mistakenly perceive a group norm and adjust their behaviour to match this assumed, rather than actual, norm [29]. As a result, individuals may act in ways that do not reflect their own beliefs. For example, they may adopt undesirable behaviours because they incorrectly believe these behaviours are widely accepted or they may remain silent because they assume others support or tolerate them [30]. In the context of GESI violations on social media, identifying these misperceptions is essential to ensure that interventions are based on actual social norms rather than distorted perceptions. However, the way these misperceptions emerge and influence behaviour may differ across cultural contexts.

In recent studies, scholars have employed a cross-cultural lens to examine the differences in online behaviour influenced by cultural diversity [31,32]. The studies explore a range of online behaviours, including social comparison [31] and users' privacy decisions [33]. While emerging studies are beginning to focus on Arab cultures and prosocial behaviour on social media [34], the majority of research has predominantly involved WEIRD (Western, educated, industrialized, rich and democratic) populations, a pattern that has been recognized for some time. The UK and Arab GCC contexts were selected for two reasons. Theoretically, they represent contrasting positions on Hofstede's individualism–collectivism dimension, making them well-suited for examining how cultural orientation shapes misperceptions around social correction. Practically, both contexts have active and growing social media populations yet remain under-represented in cross-cultural research on GESI behaviour online, particularly in comparison with each other.

Hofstede's cultural dimensions [35] highlight significant behavioural differences in social interaction between collectivist cultures, such as those in the Arab world, and individualist cultures, such as in the UK. Focusing on addressing interpersonal conflicts, Elsayed-EkJiouly & Buda [36] found that executives from the Arab Middle East often favour integrating and avoiding methods, while US executives were more likely to use approaches focused on obliging, dominating or compromising. Additionally, legal and societal frameworks influence these behaviours, for instance the UK's strong support for free speech fosters open debate [37], whereas stricter speech regulations in some Arab countries may restrict public criticism [38]. Media portrayal of violations on social media may also differ between the two cultures.

This study makes three contributions to the existing literature. First, it extends research on social norms and misperceptions by examining them specifically in the context of GESI violations on social media, an area that has received comparatively little attention. Second, it advances understanding of pluralistic ignorance online by identifying how misperceptions around social correction manifest differently across individualist and collectivist cultures. Third, it offers practical insights for platform designers and policymakers by demonstrating how social norm nudges could be used to correct misperceived norms and encourage more active challenging of GESI violations.

## 3. Material and methods

### 3.1. Participants and procedure

This study employs a cross-sectional online survey design. We developed the survey using SurveyMonkey (https://www.surveymonkey.com), a platform specialized in designing and managing online questionnaires. We reached participants from both Arab and UK countries via TGM Research services (https://tgmresearch.com), a dedicated platform for research data collection. TGM recruited participants from its existing panel database and administered an eligibility screening prior to survey access, ensuring only those meeting the inclusion criteria could proceed. The UK sample included participants from Northern Ireland, Scotland, Wales and England, while the Arab sample focused on individuals from Gulf Cooperation Council (GCC) countries, including Saudi Arabia, the United Arab Emirates, Kuwait, Qatar, Oman and Bahrain.

To include participants in the study, they should be non-passive users of social media and online forums, aged 18 or older, and culturally identify with either the UK or Arab GCC norms and culture. Participants from the GCC should be proficient in Arabic, while participants from the UK should be proficient in English. Additionally, participants must be familiar with and indicate their agreement with GESI concepts. Hence, participants were asked to read and indicate their agreement with three statements related to gender equality and three statements related to social inclusion [39]. The statements for gender equality are as follows: (i) women have the right to be represented and participate in decision-making; (ii) women have the right to equitable human development; and (iii) the recognition of cultural and linguistic diversity is an essential right for all women. The statements for social inclusion are as follows: (i) all individuals have the right to be represented and participate in decision-making; (ii) all individuals have the right to equitable human development; and (iii) the recognition of cultural and linguistic diversity is an essential right for all individuals. Participants who did not agree with all six statements were automatically redirected out of the survey and excluded from the study.

This study is part of a larger survey that explores additional questions and topics related to participants' coping responses when witnessing GESI violations, their attitudes towards gender equality and personal factors such as sense of belonging, religiosity, self-construal, self-esteem, self-efficacy and sense of power. The complete dataset and survey design can be accessed through the Open Science Framework link provided in the supplementary material. We employed multiple iterations to ensure clarity in the survey items. Initially, the survey was crafted in English. Subsequently, it underwent translation into Arabic using the back-translation method [40]. To ensure clarity and address any potential ambiguities, a trial run was carried out with a small group prior to the full launch of the survey.

Data collection commenced after obtaining ethical approval from the Institutional Review Board (IRB) of the last author's institution. Participants provided informed consent and were informed that they could withdraw from the survey at any point. The survey commenced with an introductory section outlining the study's objectives and defining the GESI concept. Eligible participants received compensation from TGM. The final dataset comprised 325 participants from the UK and 312 from Arab countries.

### 3.2. Measures

#### 3.2.1. Demographic data

We asked participants to report their gender, age, education level and marital status.

#### 3.2.2. Self-report and perception of others

To ensure clarity and consistency in responses, we provided participants with a definition of acquaintance and ensured they understood that their responses should not pertain to close friends or family members, nor to individuals who are too distant from them. Particularly, we provided participants with the following definition: ‘Acquaintance refers to connections in the broad social space between strangers and close friends on social media, such as former co-workers, neighbors, or individuals known through social media groups’. We asked the participants to confirm that they understood the definition.

To set up the scene for asking the questions related to self-reported attitudes towards challenging others who violate GESI on social media, we provided the participants with the following scenario: an acquaintance from your social media network thinks that you have shared a post that presents a violation of gender equality or social inclusion and advises you, in a way that others can see, to rethink it. An example of the self-reported attitude statement is ‘My acquaintances’ comments would offend me'. Considering the questions related to participants' perceptions of others' attitudes when those others are challenged for violating GESI principles, we provided the participants with a scenario as follows: you think that an acquaintance from your social media network has shared a post that presents a violation of gender equality or social inclusion, and you advise this acquaintance, in a way that others can see, to rethink it. A sample of the perceived attitude questions is ‘My comment would offend them’. We asked the participants to answer questions related to three folds: (i) Relationship Consequences, (ii) Negative Impact on the Person Being Challenged and (iii) Futility. We used a six-point Likert scale to measure respondents' attitudes. The scale includes six options: Strongly Disagree (i), Disagree (ii), Somewhat Disagree (iii), Somewhat Agree (iv), Agree (v) and Strongly Agree (vi). The survey items used in this study were adapted from validated scales as used in Gurgun *et al.* [41]. The questions for self-report attitudes and perceptions of others' attitudes are presented in appendix A.

To maintain data integrity, attention checks were embedded within the survey. We excluded participants who did not pass these checks or completed the survey in a time deemed too brief, that is completed in less than half of the median completion time, calculated after excluding those who took twice of more of the expected time, mostly owing to inactivity while filling the survey. Before conducting the analyses, a data quality check was performed, which involved evaluating the distribution of the data.

### 3.3. Analysis

To gain a comprehensive understanding of the data and the characteristics of the sample, descriptive statistical analyses were conducted. These analyses provided an overview of key variables such as demographics, frequencies and central tendencies.

Next, we examined the relationships between all items measuring Relationship Consequences, Negative Impact on the Person Being Challenged and Futility for both self and others. This analysis provided insights into the overall relationship between these variables and informed our decision regarding the analytical approach for modelling the data.

We employed linear mixed modelling (LMM) with a random effect of a subject to test the effects of attitudes (Relationship Consequences, Negative Impact and Futility) and person (self and other) on the rating. LMM offers several advantages over traditional analyses by incorporating both fixed and random effects [42]. It is particularly useful when data observations are correlated, such as in repeated measures or hierarchical data. By accounting for both fixed effects (predictors of interest) and random effects (e.g. individual differences), LMM effectively manages repeated measures and nested structures, enhancing statistical power by reducing unexplained variance. This robustness makes it efficient in detecting true effects, even in complex designs with repeated measures. Additionally, LMM relaxes the assumption of sphericity, allowing more flexibility regarding the independence of observations, which strengthens the analysis of repeated or nested data [43,44].

To test whether participants' ratings differed between self-reported attitudes and perceived attitudes of others and whether this difference varied across attitude types, we used LMM. The dependent variable was the item-level rating score on the six-point Likert scale, coded from 1 (Strongly Disagree) to 6 (Strongly Agree).

Separate LMMs were fitted for the Arab GCC and UK samples. In each model, Person, Attitude type and the Person × Attitude type interaction were entered as fixed effects. Person had two levels: self-reported attitude and perceived attitude of others. Attitude type had three levels: Relationship Consequences, Negative Impact on the Person Being Challenged and Futility. Participant ID was included as a random intercept to account for repeated ratings from the same respondent.

The model included fixed effects for Person, Attitude type and the Person × Attitude type interaction. Person distinguished between self-reported attitudes and perceived attitudes of others. Attitude type distinguished between Relationship Consequences, Negative Impact on the Person Being Challenged and Futility. The interaction term was included to estimate whether the difference between self-reported and perceived-other ratings changed across the three attitude types. Fixed effects were evaluated using *F*-tests. Significant effects were followed up using estimated marginal means. Pairwise contrasts were reported as mean differences between estimated marginal means, with standard errors, test statistics, adjusted *p*-values and confidence intervals. Bonferroni correction was applied to adjust for multiple comparisons.

To test whether the attitude-level effects were attributable to specific questionnaire items, we fitted a second set of LMMs separately for each sample. In these models, Person, Item and the Person × Item interaction were entered as fixed effects, with Participant ID again included as a random intercept. The Person × Item interaction tested whether self–other differences varied across individual items. Follow-up comparisons based on estimated marginal means compared self-reported and perceived-other ratings for each item, with Bonferroni adjustment applied for multiple testing. All LMMs and post hoc comparisons were conducted in jamovi 2.7.37.

## 4. Results

### 4.1. Demographic data

The demographic characteristics of both samples, including age, gender, education and marital status distribution, are presented in table 1.

**Table 1.** Demographic characteristics of UK (*n* = 325) and Arab GCC (*n* = 312) samples.

		**UK**		**Arab**	
**variable**	**item**	***f***	**%**	***f***	**%**
age group (years old)	18–30	64	19.69	120	38.46
	31–40	78	24.00	125	40.06
	41–50	82	25.23	59	18.91
	51–60	101	31.08	8	2.56
gender	female	208	64.00	170	54.49
	male	114	35.08	142	45.51
	others	3	0.92	0	0.00
education	no formal education	3	0.92	0	0.00
	primary education (elementary)	0	0.00	2	0.64
	secondary education (high school)	91	28.00	58	18.60
	undergraduate degree (bachelor's, in progress or completed)	122	37.54	210	67.31
	vocational or technical education (in progress or completed)	63	19.39	20	6.41
	postgraduate degree (master's, Ph.D. etc., in progress or completed)	46	14.15	22	7.05
marital status	married/have partner	207	63.69	200	64.10
	single	111	34.15	104	33.33
	other	7	2.15	8	2.56

*Note*: *f* = frequency (raw count); % = percentage of the total sample.

### 4.2. Descriptive statistics

Table 2 presents the descriptive statistics for both samples across all self-reported and perceived items. On average, GCC participants rated Negative Impact higher for perceived others than for themselves, while Futility and Relationship Consequences were rated higher for self than for perception of others. In the UK sample, both Futility and Relationship Consequences were rated higher for perceived than for self, while Negative Impact was rated higher for self than for perception of others. Finally, the variables in both samples displayed skewness and kurtosis within the range of −2 to +2, which is considered acceptable [45].

**Table 2.** Descriptive statistics for GCC and UK samples.

	GCC		UK			GCC		UK	
self-reported	mean	s.d.	mean	s.d.	perceived	mean	s.d.	mean	s.d.
**Relationship Consequences (RC)**									
RC-1	3.718	1.51	3.394	1.335	RC-1	3.34	1.5	3.84	1.149
RC-2	3.256	1.467	3.329	1.284	RC-2	3.346	1.397	3.609	1.206
RC-3	3.41	1.454	3.557	1.401	RC-3	3.372	1.385	3.745	1.249
RC-4	3.359	1.463	3.458	1.375	RC-4	3.327	1.397	3.471	1.238
RC-5	2.865	1.486	3.505	1.413	RC-5	3.006	1.33	3.76	1.254
RC-6	3.359	1.521	3.714	1.453	RC-6	3.542	1.427	3.895	1.258
**AVG (RC)**	**3.328**	**1.289**	**3.493**	**1.226**	**AVG (RC)**	**3.322**	**1.188**	**3.72**	**1.074**
**Negative Impact on the Person Being Challenged (NI)**									
NI-1	3.724	1.505	4.142	1.372	NI-1	3.942	1.357	3.972	1.101
NI-2	3.462	1.569	3.791	1.338	NI-2	3.676	1.405	3.695	1.177
**AVG (NI)**	**3.593**	**1.45**	**3.966**	**1.255**	**AVG (NI)**	**3.809**	**1.309**	**3.834**	**1.06**
**Futility (*F*)**									
*F*-1	3.413	1.557	3.065	1.307	*F*-1	3.33	1.345	3.548	1.115
*F*-2	3.538	1.645	3.08	1.44	*F*-2	3.481	1.363	3.631	1.074
**AVG (*F*)**	**3.476**	**1.489**	**3.072**	**1.193**	**AVG (*F*)**	**3.405**	**1.219**	**3.589**	**0.988**

To assess internal consistency in the present samples, we computed Cronbach's alpha for each subscale (Relationship Consequences, Negative Impact and Futility), separately for the self-reported and perceived-other versions, in both the UK and Arab GCC samples. Relationship Consequences (*α* = 0.919–0.947) and Negative Impact (*α* = 0.834–0.886) showed good to excellent reliability across both samples and versions. Futility showed acceptable reliability overall (*α* = 0.671–0.843), consistent with the known instability of two-item alpha estimates for shorter subscales.

We also analysed the relationships between all items in each sample separately using Pearson correlation (see appendix B). The results showed effect sizes ranging from small to large, indicating non-independence among the items. Since these items are nested within the three attributes (Relationship Consequences, Negative Impact on the Person Being Challenged and Futility) and within each participant by design, we used LMM to account for this non-independence.

### 4.3. LMMs testing the effects of attitudes for self and others

The analysis was conducted in two steps. First, to gain an overall understanding of participants' ratings on the attitudes (Relationship Consequences, Negative Impact on the Person Being Challenged and Futility) for both self and others, as well as to assess interactions between these variables, we performed an LMM for each sample (table 3). Second, for a more detailed analysis, we examined the effects of individual items and compared the ratings between self and perceived others using a separate LMM. In each LMM model, we entered two factors as independent variables: Person (self and perceived) and Attitudes (Relationship Consequences, Negative Impact on the Person Being Challenged and Futility). To account for between-subject variability, we entered a participant's ID as a random effect.

**Table 3.** Omnibus LMM tests for Person, Attitude type and their Interaction.

**sample**	**effect**	**df1**	**df2**	***F***	***p***
**Arab GCC**	Person	1	5923	2.05	0.152
	Attitude type	2	5923	52.02	<0.001
	Person × Attitude type	2	5923	5.94	0.003
**UK**	Person	1	6170	52.97	<0.001
	Attitude type	2	6170	107.06	<0.001
	Person × Attitude type	2	6170	35.04	<0.001

In the GCC cohort, there was no significant effect of Person, indicating that overall ratings did not differ between self-reported attitudes and perceptions of others' attitudes. There was a significant effect of Attitudes, driven by lower ratings for Futility compared to Negative Impact, higher ratings for Futility compared to Relationship and higher ratings for Negative Impact compared to Relationship (all *p*_bonf_ ≤ 0.005; see table C1 in appendix C for full statistics). There was also a significant interaction between Person and Attitudes, driven by significantly higher ratings for Negative Impact for others compared to the self, while the differences between self and other for Futility and Relationship were non-significant (see appendix C and figure 1).

**Figure 1.**
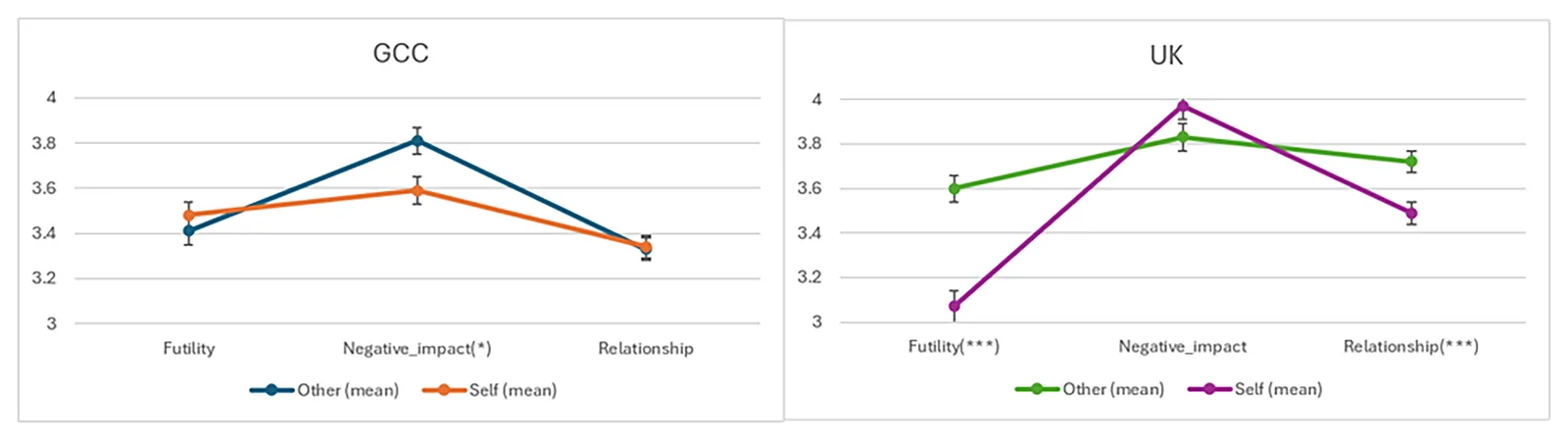
Estimated marginal means for interaction between Attitudes and Person across Arab and UK samples. Error bars represent a 95% confidence interval, *p < 0.05, **p < 0.01, ***p < 0.001, ns ≥ 0.05.

In the UK cohort, there was a main effect of Person, with ratings for others higher than for the self. There was also a main effect of Attitudes, with Futility rated lower than Negative Impact and Relationship and Negative Impact rated higher than Relationship (all *p*_bonf_ < 0.001; see table C2 in appendix C). We also found an interaction between Person and Attitudes: in contrast to the GCC cohort, this was driven by significantly lower ratings for self compared to other for Futility and Relationship, with no difference between self and other for Negative Impact (see appendix C and figure 1). These findings address RQ1 by showing that self-reported attitudes and perceptions of others' attitudes diverge for the Negative Impact attitude in the GCC sample, while diverging for Futility and Relationship Consequences in the UK sample.

The standardized effect sizes presented in figure 2 further demonstrate that the magnitude of the self-other discrepancies differed across attitudes and samples. In the Arab GCC sample, the discrepancy for Negative Impact represented a small positive effect, whereas the effects for Futility and Relationship Consequences were negligible. In the UK sample, the discrepancy for Futility represented a moderate positive effect, and that for Relationship Consequences represented a small positive effect. The effect for Negative Impact was small and negative, but its adjusted confidence interval included zero.

**Figure 2.**
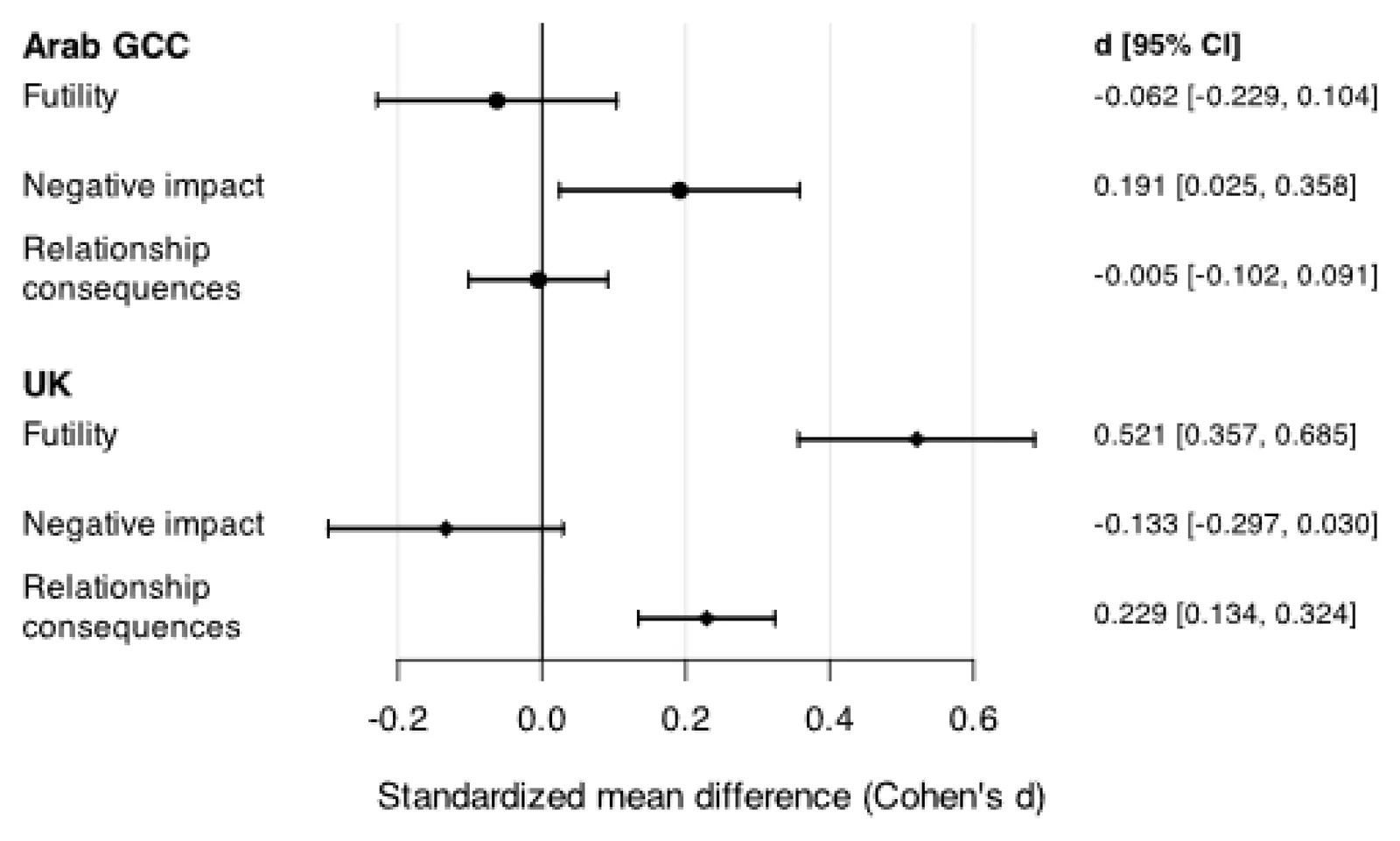
Standardized effect sizes for self–other differences across attitude types in the Arab GCC and UK samples. Points represent model-based Cohen's *d* values, calculated by dividing each estimated LMM contrast by the model residual standard deviation. Contrasts were calculated as perceived-other minus self-report; therefore, positive values indicate higher perceived-other ratings, and negative values indicate higher self-reported ratings. Error bars represent Bonferroni-adjusted 95% confidence intervals based on the 15 pairwise comparisons among the six Person × Attitude type combinations. Circles represent the Arab GCC sample, and diamonds represent the UK sample.

#### 4.3.1. LMM testing the effects of items and comparing them between self and the perception of others

Figure 1 illustrates the differential effects of attitudes across self and perceived in both the GCC and UK cohorts. To explore these effects in detail, we carried out an LMM analysis, testing the effects of items on ratings and using post hoc comparisons with adjustments for multiple comparisons to compare the items between self and others in each cohort.

In both cohorts, there was a significant main effect of Item, indicating that different items had varying effects on ratings (*F*(19,5909) = 14.5, *p* < 0.001 for the GCC sample; *F*(19,6156) = 24.8, *p* < 0.001 for the UK sample). To clarify these effects, we performed post hoc comparisons between all items; here, we focus on comparisons that are directly relevant to our research questions.

Table 4 shows that, once corrected, the item-level self-other discrepancy in the GCC sample is not driven by the Negative Impact item as previously reported; instead, only the first Relationship Consequences item (RC-1) showed a significant self-other difference, with perceived-other ratings lower than self-reported ratings ( *t* = −4.23, *p*_bonf_ = 0.005). None of the Futility or Negative Impact items differentiated self from other ratings in the GCC sample. In the UK sample, by contrast, both Futility items and the first Relationship Consequences item showed significant self-other differences, with others rated significantly higher than self on Futility (*t* = 6.25 and *t* = 7.13, both *p*_bonf_ < 0.001) and on RC-1 (*t* = 5.78, *p*_bonf_ < 0.001). Thus, the pattern of item-level discrepancies differs qualitatively between samples: in the UK sample, the self-other gap is concentrated in the Futility items, whereas in the GCC sample it is confined to a single Relationship Consequences item, with no evidence of a Negative Impact–driven discrepancy. These item-level results address RQ2: the self-other discrepancy is different between the two cultural contexts.

**Table 4.** The results of the post hoc test comparing items between self and perceived attitudes in the GCC and UK samples.

attitudes	pairs	Arab (*n* = 312)	UK (*n* = 325)
	self-reported vs. perceived	*t*-test (df = 5909), *p*_bonf_	*t*-test (df = 6156), *p*_bonf_
Relationship Consequences	RC-1	*t* = −4.23**	*t* = 5.78***
	RC-2	*t* = 1.00	*t* = 3.62
	RC-3	*t* = −0.43	*t* = 2.43
	RC-4	*t* = −0.35	*t* = 0.16
	RC-5	*t* = 1.58	*t* = 3.31
	RC-6	*t* = 2.04	*t* = 2.35
Negative Impact	NI-1	*t* = 2.44	*t* = −2.19
	NI-2	*t* = 2.40	*t* = −1.23
Futility	*F*-1	*t* = −0.93	*t* = 6.25***
	*F*-2	*t* = −0.65	*t* = 7.13***

*Notes*: *p < 0.05, **p < 0.01, ***p < 0.001.

The effect sizes (figure 3) show that the Arab GCC Negative Impact estimates were nearly identical for NI-1 and NI-2, meaning that the attitude-level finding was reflected across both items despite neither item reaching significance after correction. In the UK, the Futility estimates were larger and similarly consistent across *F*-1 and *F*-2. Thus, the effect-size results distinguish a weaker but consistent Negative Impact pattern in the Arab GCC sample from a stronger Futility pattern in the UK sample.

**Figure 3.**
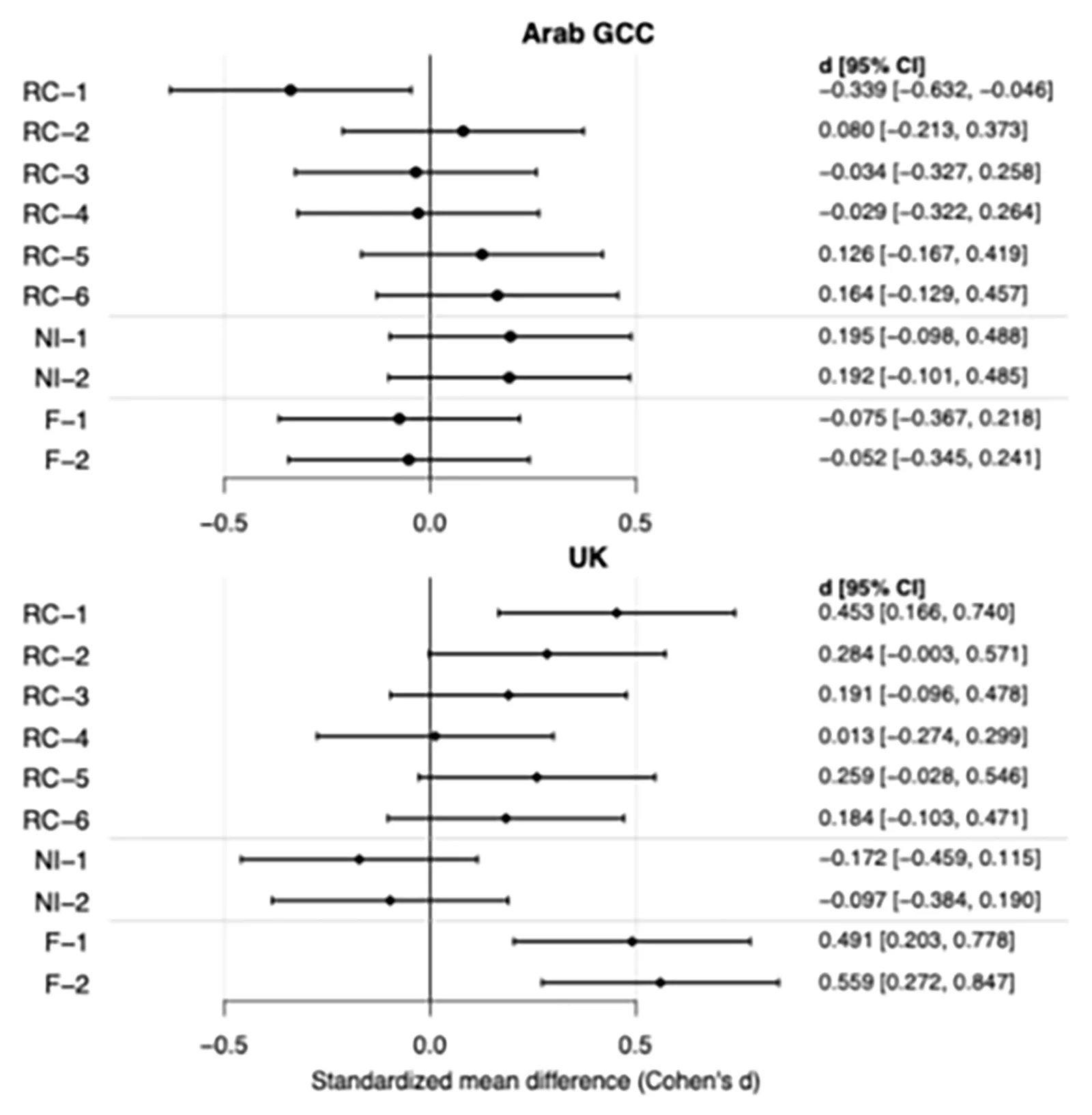
Standardized effect sizes for item-level self–other differences in the Arab GCC and UK samples. Points represent model-based Cohen's *d* values, calculated by dividing each estimated LMM contrast by the model residual standard deviation. Contrasts were calculated as perceived-other minus self-reported; therefore, positive values indicate higher perceived-other ratings, and negative values indicate higher self-reported ratings. Error bars represent Bonferroni-adjusted 95% confidence intervals. RC = Relationship Consequences; NI = Negative Impact; F = Futility.

In addition, we analysed the probability of participant rating for Attitudes for self and others (figure 4). The UK sample exhibits greater uncertainty, with higher probability values at mid-range ratings (3 and 4) compared to the GCC group, particularly in perceived variables. This suggests that UK respondents are more inclined to somewhat agree or somewhat disagree with the statements, indicating a higher level of indecision or ambiguity. Furthermore, the probability for the UK sample decreases at higher ratings (5 and 6). In contrast, the GCC group displays greater certainty, with higher probability values at the higher ratings, suggesting that GCC respondents are more likely to agree or strongly agree with the statements, reflecting a more confident perspective. This pattern in response style is descriptive rather than a tested difference in attitudes and does not indicate that GCC respondents view corrective behaviour as more or less likely to succeed.

**Figure 4.**
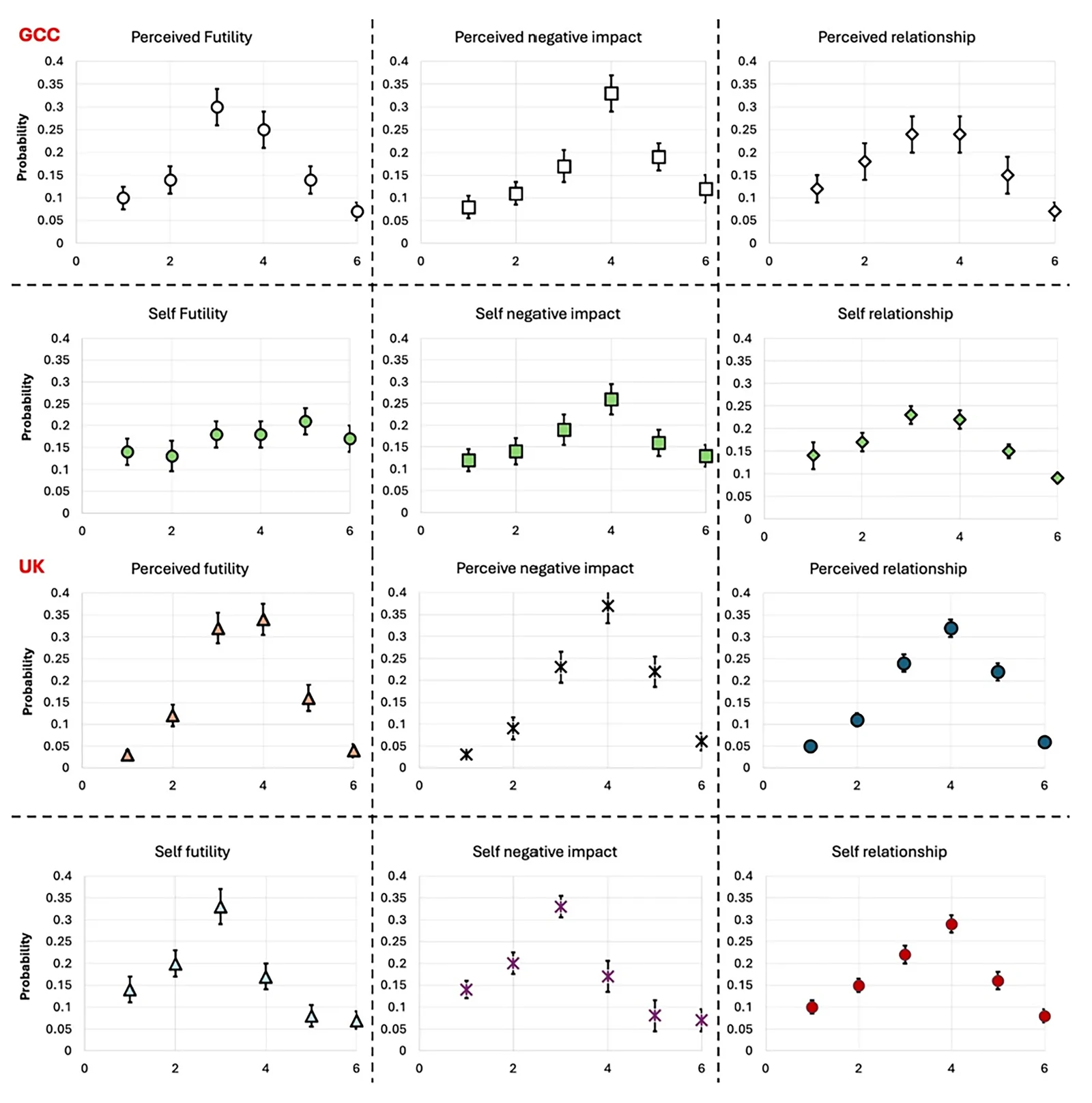
Probability of participants rating in the GCC and UK samples. Error bars represent the 95% confidence interval.

## 5. Discussion

This study examined users' self-reported attitudes and their perceptions of others' attitudes towards challenging GESI violations on social media, focusing on three key barriers: Relationship Consequences, Negative Impact on others and Futility. While these factors have been investigated in related contexts such as confronting misinformation [32,46], comparatively little attention has been paid to how they shape responses to GESI violations specifically.

Addressing RQ1, the findings reveal a discrepancy between self-reported attitudes and perceptions of others across both samples, though, as detailed below, the nature of these misperceptions differs by cultural context. This may be explained by pluralistic ignorance, whereby individuals infer that their own attitudes and actions stem from internal states or motives that differ from those underlying the same attitudes and actions in others, leading people to perceive their own views as diverging from others' even when they may not [47]. Referring to RQ1.1, RQ1.2 and RQ1.3, figure 1 shows the interaction between Attitudes and Person across Arab and UK samples. At the attitude level, misperceptions in the UK were present in two factors: Relationship Consequences and Futility, while misperceptions in the GCC were present in one factor, Negative Impact on others.

Turning to RQ2, these misperceptions manifested differently across the two cultural contexts, which we interpret through Hofstede's individualism–collectivism dimension [48]. Arab societies are generally defined by collectivism, while the UK is characterized by individualism [35]. In individualist communities, such as the UK, individuals express more self-esteem in the presence of others [49]. This may explain why UK participants believed that an acquaintance they confronted would be less willing to change their opinion than they themselves would be if the situation were reversed. It may also explain why they expected confronting someone else to cause more damage to the relationship than being confronted themselves. These findings suggest that participants had greater confidence in their own ability to accept criticism and reconsider their views than they attributed to others.

This discrepancy may also reflect the well-documented tendency towards self-enhancement in individualist cultures [50]. People in these cultures are more likely to view themselves positively. Other features of individualism, including competition, individual achievement, self-reliance and autonomy, further explain these dynamics [51]. This tendency is closely associated with the independent self-construal that characterizes individualist societies and is generally less pronounced in collectivist cultures [47]. This may explain both UK-specific patterns observed in the present study. For Futility, UK participants may have viewed themselves as more able to respond constructively to criticism and revise their views, while assuming that a confronted acquaintance would be less willing or able to do so. For Relationship Consequences, participants may likewise have perceived themselves as more capable of coping with interpersonal disagreement without causing lasting harm to the relationship, while assuming that an acquaintance would be less resilient and that the relationship would be more negatively affected.

In the Arab GCC sample, the results indicated that participants perceived the person being challenged as more negatively affected than they believed they themselves would be, though this pattern was not consistent across the individual Negative Impact items. At the item level, the significant self-other discrepancy was instead concentrated in the first Relationship Consequences item (RC-1), which asks whether one's comment would offend the person being challenged. This pattern is consistent with face-negotiation theory, which holds that individuals from collectivist cultures tend to show greater concern for the other party's ‘face’ than for their own, whereas individualist cultures show the reverse pattern [52,53]. This concern for the other's face often extends beyond one's own image to actively protecting the face of the person one is interacting with, particularly within one's in-group [54]. While the cultural dimension offers a useful interpretive lens, we acknowledge that cultural dimensions are broad constructs and that other factors, such as platform usage patterns, legal frameworks and individual variation, may also contribute to the observed differences.

Referring to figure 4, the comparison between GCC and UK participants reveals differences in certainty when assessing challenging behaviour considering Futility, Negative Impact and Relationship Consequences for both self-reported and perceived attitudes. UK respondents tend to be more uncertain in their evaluations, often taking moderate attitudes (higher probability values at mid-range ratings), while Arab GCC respondents expressed strong agreement with the statements, indicating a higher level of certainty in their views (higher probability values at the higher ratings). This pattern may tentatively suggest that GCC respondents are somewhat more sensitive to the impact of challenging behaviour on their relationships and others and could point to a perception among GCC individuals that changing the violator's behaviour is less probable compared to those in the UK. As noted above, however, this remains a descriptive pattern rather than a confirmed attitudinal difference and should be interpreted cautiously. The study's results are consistent with previous literature, given that Arabs are considered to be part of a collectivist culture [55]. Collectivism reflects an individual's tendency to value being part of a tightly knit social group over pursuing personal interests and acting independently [56]. Collectivism frequently results in cautious and restrained behaviour, especially when engaging with those outside of the immediate social group [56].

### 5.1. Research implications

This study makes several theoretical and empirical contributions. Theoretically, it extends research on pluralistic ignorance by demonstrating that misperceptions around social correction of GESI violations are present in both individualist and collectivist cultural contexts, yet manifest differently across them. This advances understanding of how cultural orientation shapes the gap between private attitudes and perceived social norms online. Empirically, it provides cross-cultural evidence, comparing UK and Arab GCC samples, on three specific barriers to challenging GESI violations: Relationship Consequences, Negative Impact on others and Futility. To our knowledge, this is one of the first studies to examine misperceptions around social correction of GESI violations specifically, rather than misinformation or other online harms more broadly.

Social media platforms operate within an attention-based economy, where the design features and algorithms are strategically developed to capture and sustain user engagement [57]. These platforms generate revenue by selling advertising space to companies, which is determined by how much attention users give to content. This business model creates a skewed incentive structure in which surprising, extreme or divisive content is disproportionately rewarded, sometimes at the cost of accuracy or ethical considerations [58]. As a result, the most active users tend to produce more sensational content to maintain visibility and engagement [10,59]. For example, one large-scale study of over 2.7 million posts found that each additional word referencing a political out-group increased the odds of a post being shared by around 67% [60]; a dynamic that plausibly extends to hostility towards other social out-groups, including those targeted by GESI violations. This business model may also reinforce the cycle of pluralistic ignorance, as individuals could reasonably conclude that such extreme views are more widely accepted than they actually are. As a result, users may overestimate the general acceptance of such behaviour, reducing their likelihood to challenge it. Understanding this misperception highlights the need for social media platforms to consider interventions that promote accurate social norms around challenging GESI violations. To address this issue, social media platforms should consider redesigning their algorithms and incentive structures to promote civility and constructive engagement rather than prioritizing popularity or virality.

The findings of the study have several important implications, particularly underscoring the need to address pluralistic ignorance to encourage behavioural change. By addressing pluralistic ignorance, we can foster more inclusive online environments and encourage users to take action against discriminatory behaviour, rather than passively accepting or overlooking such violations. The effectiveness of addressing pluralistic ignorance is supported by the literature in various contexts. The research by Schroeder and Prentice [61] on student alcohol use illustrates how addressing pluralistic ignorance can lead to significant behavioural changes. This was achieved by correcting students' misperception that their peers were more comfortable with heavy drinking than they actually were; as a result, the authors reduced alcohol consumption on campus. The study by Buzinski *et al*. [29] indicated that the impact of pluralistic ignorance could be reduced by explaining the real social norm and offering people correct information about the actual norm that exists.

Our findings highlight the importance of implementing a strategy to reduce pluralistic ignorance on social media by addressing misperceived norms through targeted interventions within the platform design. Among these interventions, social norm nudges can be employed to combat online toxicity and further address misperceptions. These nudges can be integrated as a simple design element in the platform [62] or as a message presented to the user [63]. Building on the work by Cialdini *et al*. [64], descriptive norms refer to the common or typical behaviours observed in a particular context, while injunctive norms relate to the expectations or beliefs about behaviours that are considered morally acceptable or unacceptable. Both types of norms have been incorporated into the design of nudging strategies in previous studies aimed at promoting behaviour change [65]. Incorporating such social norm nudges into platform design can clarify the actual norms of others, particularly concerning responses to GESI violations. For example, if users underestimate the frequency of others correcting negative behaviours, interventions should emphasize the actual rate of these corrective actions. This method can adjust misperceptions and cultivate a more positive and inclusive online space.

### 5.2. Limitations and future work

Several limitations must be taken into consideration in interpreting our findings and guiding future studies. First, the sample's representativeness may be limited, as most Arab participants are from Saudi Arabia and most British participants are from England. Although this focus allows for detailed insights within these specific groups, it may not fully capture the cultural diversity across the wider Arab GCC and British populations. Future research could benefit from including participants from additional regions to improve cross-cultural applicability. Consequently, the theoretical interpretations offered above should be read as most directly applicable to these specific national contexts, rather than to the Arab GCC and UK populations more broadly. Second, requiring participants to agree with all six GESI statements was a deliberate choice, not an oversight: we needed participants who already understood and supported these principles before asking them about challenging violations of them. Combined with recruiting through TGM Research's panel, this means our findings tell us how people who already support GESI think about social correction, not how people who are unsure about or opposed to GESI would respond, nor how the general population of the UK and Arab GCC would respond more broadly. Third, excluding individuals under 18 restricts the study's ability to capture the perspectives of younger age groups, who may approach challenging GESI violations differently. Fourth, the reliance on self-reported data also presents potential biases, such as social desirability and limitations in accurate self-assessment. To help minimise these biases, we incorporated anonymous data collection and carefully designed the survey questions to reduce leading language and use neutral phrasing. Finally, adding qualitative data in future research could facilitate our understanding of personal and contextual factors shaping attitudes towards social correction of GESI violations.

## 6. Conclusion

This study investigated misperceptions around social correction of GESI violations on social media, focusing on three barriers: Relationship Consequences, Negative Impact on others and Futility. Findings reveal that misperceptions exist in both the UK and Arab GCC contexts, though they manifest differently. UK participants perceive greater relational costs and lower odds of behavioural change when imagining themselves confronting others than when imagining being confronted themselves. In the GCC sample, participants perceived the person being challenged as more negatively affected than they believed they themselves would be, though this pattern was less consistent across individual measures and warrants further investigation. The study contributes to the literature by providing cross-cultural evidence on how pluralistic ignorance shapes responses to GESI violations online. Practically, the findings highlight the potential of social norm nudges embedded in platform design to correct misperceived norms, reduce barriers to social correction and foster more inclusive online environments.

## Figures and Tables

**Table A1.** Survey questions for self-reported and perceptions of others' attitudes.

	**self-reported**		**perceptions of others**	
variables	abbreviations	questions	abbreviations	questions
Relationship Consequences (RC)	self-reported (RC-1)	my acquaintances' comments would offend me	perceived (RC-1)	my comment would offend them
	self-reported (RC-2)	my acquaintances' comments could make me see them as being aggressive	perceived (RC-2)	my comment would make them think that I am aggressive
	self-reported (RC-3)	my acquaintances' comments could make me see them as being unfriendly	perceived (RC-3)	my comment would make them think that I am unfriendly
	self-reported (RC-4)	my acquaintances' comments could make me see them as being unempathetic	perceived (RC-4)	my comment would make them think that I am unempathic
	self-reported (RC-5)	the relationship between us will deteriorate	perceived (RC-5)	the relationship between us will deteriorate
	self-reported (RC-6)	we will interact less frequently afterward	perceived (RC-6)	we will interact less frequently afterward
Negative Impact on the Person Being Challenged (NI)	self-reported (NI-1)	I would feel embarrassed or upset	perceived (NI-1)	they would feel embarrassed or upset
	self-reported (NI-2)	I would feel that I would be viewed as untrustworthy by others	perceived (NI-2)	they would feel that they would be viewed as untrustworthy by others
Futility (*F*)	self-reported (*F*-1)	it wouldn't make a difference to change my mind	perceived (*F*-1)	it wouldn't make a difference to change his/her mind
	self-reported (*F*-2)	it wouldn't make me amend or delete the post	perceived (*F*-2)	it wouldn't make them amend or delete the post

**Figure B1.**
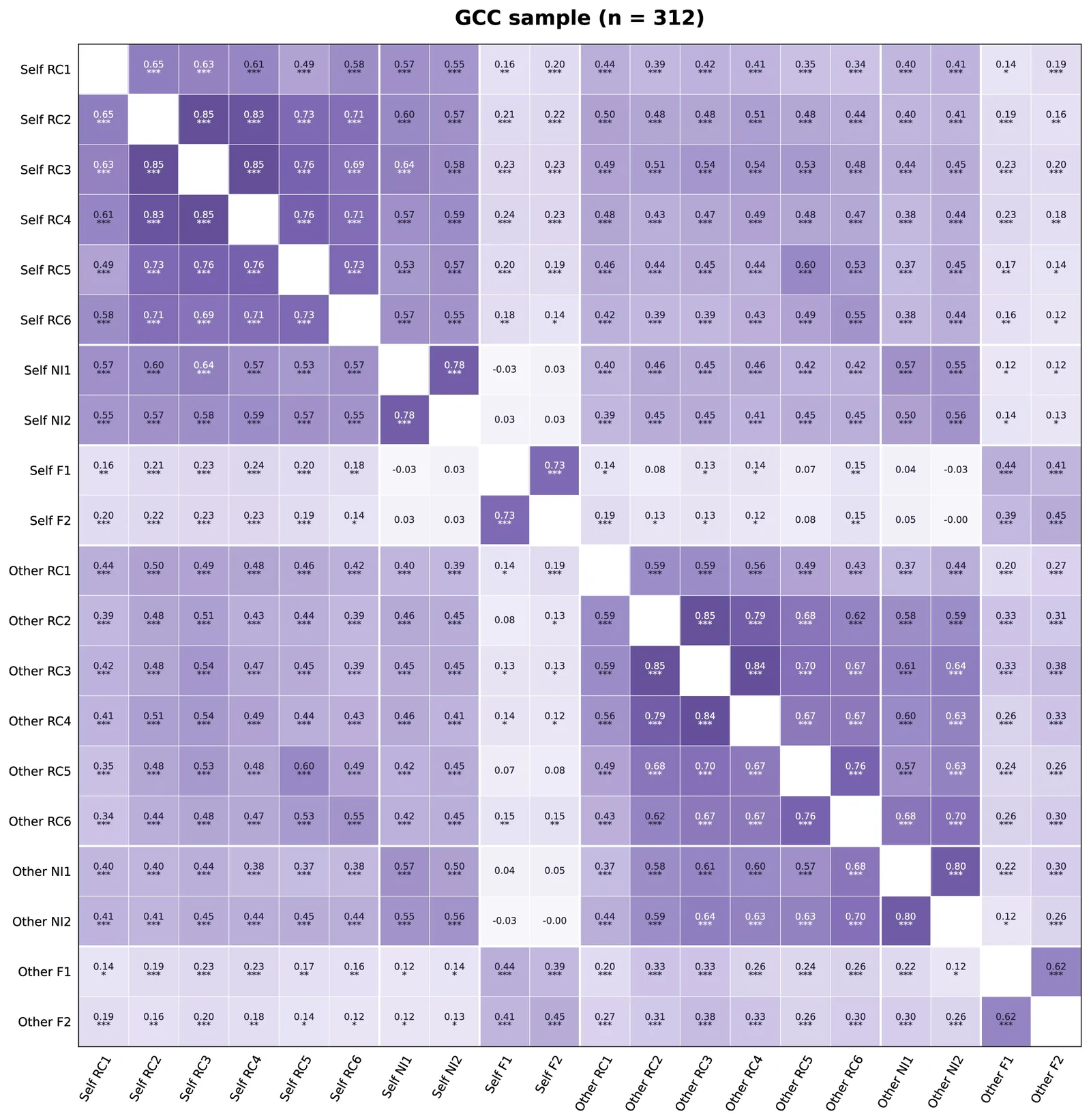
Pearson correlation between items for GCC.

**Figure B2.**
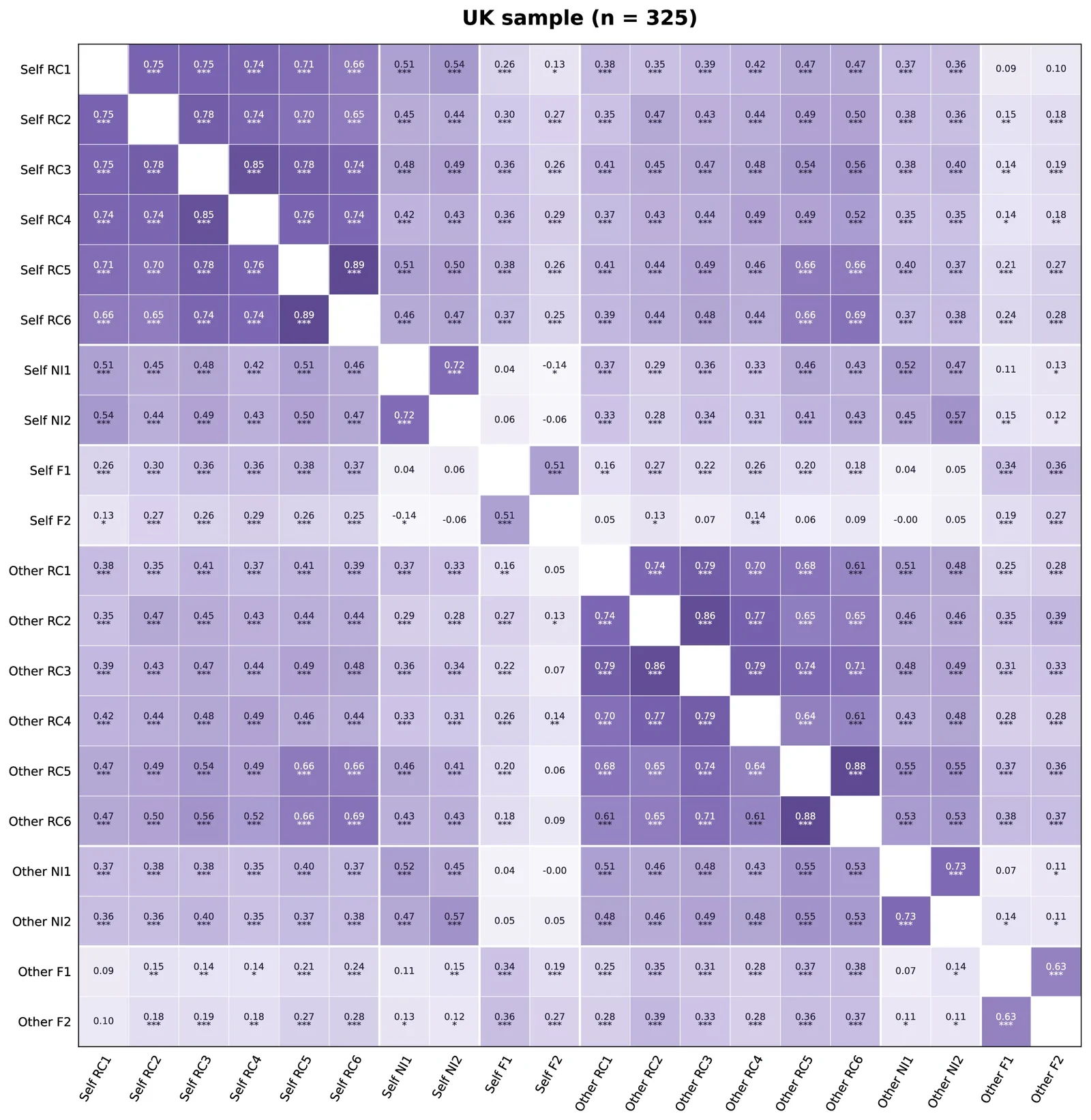
Pearson correlation between items for the UK.

**Table C1.** The results of the post hoc test comparing Person × Attitude type in the GCC (linear mixed model).

**variables**	**person**	**variables**	**person**	**difference**	**SE**	***t***	***p*_bonferroni_**
Futility	other	Futility	self	−0.07051	0.064	−1.102	1
Futility	other	Negative Impact	other	−0.40385	0.064	−6.313	<0.001
Futility	other	Negative Impact	self	−0.1875	0.064	−2.931	0.051
Futility	other	Relationship	other	0.08333	0.0522	1.595	1
Futility	other	Relationship	self	0.07746	0.0522	1.483	1
Futility	self	Negative Impact	other	−0.33333	0.064	−5.21	<0.001
Futility	self	Negative Impact	self	−0.11699	0.064	−1.829	1
Futility	self	Relationship	other	0.15385	0.0522	2.945	0.049
Futility	self	Relationship	self	0.14797	0.0522	2.833	0.069
Negative Impact	other	Negative Impact	self	0.21635	0.064	3.382	0.011
Negative Impact	other	Relationship	other	0.48718	0.0522	9.327	<0.001
Negative Impact	other	Relationship	self	0.4813	0.0522	9.214	<0.001
Negative Impact	self	Relationship	other	0.27083	0.0522	5.185	<0.001
Negative Impact	self	Relationship	self	0.26496	0.0522	5.072	<0.001
Relationship	other	Relationship	self	−0.00588	0.0369	−0.159	1

*df* = 5923 for all comparisons.

**Table C2.** The results of the post hoc test comparing Person × Attitude type in the UK (linear mixed model).

**variables**	**person**	**variables**	**person**	**difference**	**SE**	***t***	***p*_bonferroni_**
Futility	other	Futility	self	0.5169	0.055	9.39	<0.001
Futility	other	Negative Impact	other	−0.2446	0.055	−4.45	<0.001
Futility	other	Negative Impact	self	−0.3769	0.055	−6.85	<0.001
Futility	other	Relationship	other	−0.1308	0.0449	−2.91	0.054
Futility	other	Relationship	self	0.0964	0.0449	2.15	0.479
Futility	self	Negative Impact	other	−0.7615	0.055	−13.84	<0.001
Futility	self	Negative Impact	self	−0.8938	0.055	−16.24	<0.001
Futility	self	Relationship	other	−0.6477	0.0449	−14.41	<0.001
Futility	self	Relationship	self	−0.4205	0.0449	−9.36	<0.001
Negative Impact	other	Negative Impact	self	−0.1323	0.055	−2.4	0.244
Negative Impact	other	Relationship	other	0.1138	0.0449	2.53	0.17
Negative Impact	other	Relationship	self	0.341	0.0449	7.59	<0.001
Negative Impact	self	Relationship	other	0.2462	0.0449	5.48	<0.001
Negative Impact	self	Relationship	self	0.4733	0.0449	10.53	<0.001
Relationship	other	Relationship	self	0.2272	0.0318	7.15	<0.001

*Note*: df = 6170 for all comparisons.

## Data Availability

The research materials supporting this study, including the final dataset, analysis files and supplementary materials, are available on the Open Science Framework (OSF) repository [66].
